# High competing risks minimize real-world utility of adjuvant targeted therapy in renal cell carcinoma: a population-based analysis

**DOI:** 10.18632/oncotarget.24675

**Published:** 2018-03-30

**Authors:** Thenappan Chandrasekar, Zachary Klaassen, Hanan Goldberg, Rashid K. Sayyid, Girish S. Kulkarni, Neil E. Fleshner

**Affiliations:** ^1^ Department of Surgical Oncology, Division of Urology, University Health Network, University of Toronto, Ontario, Canada

**Keywords:** carcinoma, renal cell, neoplasm metastasis, drug therapy, survival, mortality

## Abstract

**Objective:**

To utilize a population-based approach to address the role of adjuvant TT in the management of RCC.

**Methods:**

Patients with RCC (2006-2013) in the SEER database were stratified by metastatic disease at the time of diagnosis (cM0/cM1). cM0 patients following surgical excision were stratified into low and high-risk (ASSURE and S-TRAC criteria). Multivariable analyses performed to identify predictors of TT receipt; Fine and Gray competing risks analyses used to identify predictors of cancer-specific mortality (CSM). Subset analyses included patients with clear cell histology and high-risk cM0. Survival analyses were used to evaluate overall survival (OS) and cancer-specific survival (CSS) for all cohorts, stratified on TT receipt.

**Results:**

79,926 patients included (71,682 cM0, 8,244 cM1); median follow-up for the entire cohort was 40.1 months. Of 31,453 patients with histologic grade data, 18,328 and 13,125 were low- and high-risk cM0, respectively. TT utilization in cM1 patients peaked at 50.6% and was associated with reduced CSM (HR 0.73, p<0.01). In contrast, TT utilization (presumed salvage therapy) never exceeded 2.2% in the entire cM0 cohort and 3.5% in the high-risk cM0 cohort. On competing risks analysis, TT receipt was associated with increased CSM in all cohorts.

**Conclusion:**

When compared to the cM1 patients, TT receipt in cM0 patients does not provide any cancer-specific survival benefit, even in the small percentage of patients that eventually progress to metastatic disease. Competing risks mortality further limit any potential benefit in this population. Based on current evidence, adjuvant TT cannot be recommended for RCC patients.

## INTRODUCTION

Renal cell carcinoma (RCC) has traditionally been surgically managed. Extirpative surgery, either radical nephrectomy (RN) or partial nephrectomy (PNx), remains the standard of care for localized disease [1, 2]. Radiation therapy and systemic therapy have not been proven to be effective for the management of localized disease.

Targeted therapies (TT), including tyrosine kinase inhibitors (TKIs) and mTOR inhibitors, were first introduced in 2006 [3–5]. Since then, they have become a cornerstone of RCC therapy, specifically for metastatic RCC [1]. Utilized in patients with de novo metastatic RCC or patients with metastatic progression following primary surgical management, they have been demonstrated to extend progression-free survival by 3-8 months in patients with clear-cell histology [1, 2, 6, 7]. Their introduction has even called into question the oncologic value of cytoreductive nephrectomy, which was first established in the cytokine era [8–11].

While the efficacy of targeted therapies is well established for metastatic RCC, its role as an adjuvant therapy is less clear. Two randomized controlled trials (RCTs) demonstrated conflicting cancer-specific survival outcomes with sunitinib and sorafenib in the adjuvant setting for high-risk localized RCC [12–15]. In ASSURE, there was no significant difference in disease-free survival (DFS) between high-risk patients treated with sunitinib, sorafenib or placebo, while in S-TRAC, sunitinib-treated patients had a 1.2 year improved DFS. Given these conflicting results, we aim to examine the utilization of TT in the targeted-therapy era. In doing so, we aim to identify predictors of TT receipt and cancer-specific survival (CSS), particularly in patients with non-metastatic localized RCC treated with definitive surgery, to further examine the role for adjuvant therapy in these high-risk patients.

## RESULTS

### Demographics

Table 1 details the demographics of the entire cohort (N=79,926), stratified by cM0 (N=71,682) or cM1 (N=8,244) at the time of diagnosis. Patients with cM1 disease were more likely to be older, male, and underinsured, while also presenting with higher cT and cN stage. Most cM0 patients underwent primary surgical intervention, while only 45.9% of cM1 patients had surgery. Median follow-up for the entire cohort was 40.1 months (± 27.7 months).

**Table 1:** Patient demographics, stratified by cM status

	cM0	cM1	p-value
**Total Number, N**	71682	8244	
**Age at Diagnosis, Mean (SD)**	61.83 (13.04)	64.31 (12.30)	<0.001
**Sex, Male (%)**	45211 (63.1)	5619 (68.2)	<0.001
**Region (%)**			
**Southeast**	12764 (17.8)	1590 (19.3)	<0.001
**Midwest**	12656 (17.7)	1454 (17.6)	
**West**	34995 (48.8)	4177 (50.7)	
**Northeast**	11267 (15.7)	1023 (12.4)	
**Insurance (%)**			
**Medicaid**	6402 (10.2)	990 (13.6)	<0.001
**Uninsured**	1905 (3.0)	323 (4.4)	
**Insured**	54519 (86.8)	5962 (82.0)	
**Marital Status (%)**			
**Single**	10280 (15.1)	1262 (15.8)	<0.001
**Divorced/Separated**	7183 (10.6)	914 (11.4)	
**Widowed**	6754 (9.9)	984 (12.3)	
**Married**	43796 (64.4)	4834 (60.5)	
**Race (%)**			
**Hispanic**	9418 (13.1)	1141 (13.8)	<0.001
**American Indian/Alaskan**	591 (0.8)	92 (1.1)	
**Asian or Pacific Islander**	3395 (4.7)	449 (5.4)	
**Black**	8315 (11.6)	797 (9.7)	
**White**	49575 (69.2)	5754 (69.8)	
**Socioeconomic Status (%)**			
**1 = Highest quartile**	13711 (19.1)	1484 (18.0)	<0.001
**2**	15617 (21.8)	1660 (20.1)	
**3**	19208 (26.8)	2339 (28.4)	
**4 = Lowest quartile**	23146 (32.3)	2761 (33.5)	
**Laterality (%)**			
**Right-sided primary**	36308 (50.7)	3888 (47.4)	<0.001
**Left-sided primary**	35288 (49.3)	4276 (52.1)	
**Bilateral**	51 (0.1)	46 (0.6)	
**Histology (%)**			
**Clear Cell RCC**	41938 (58.5)	3771 (45.7)	<0.001
**Papillary RCC**	9597 (13.4)	388 (4.7)	
**Chromophobe RCC**	4320 (6.0)	70 (0.8)	
**Sarcomatoid RCC**	555 (0.8)	490 (5.9)	
**RCC, Unspecified**	15272 (21.3)	3525 (42.8)	
**cT stage (%)**			
**cT1**	51748 (72.2)	1886 (22.9)	<0.001
**cT2**	7938 (11.1)	1612 (19.6)	
**cT3**	11437 (16.0)	3522 (42.7)	
**cT4**	559 (0.8)	1224 (14.8)	
**cN stage (%),**			
**CN1**	1345 (1.9)	2795 (33.9)	<0.001
**Fuhrman Grade (%)**			
**Grade 1**	3559 (11.2)	70 (3.3)	<0.001
**Grade 2**	17405 (54.5)	485 (23.2)	
**Grade 3**	9162 (28.7)	833 (39.9)	
**Grade 4**	1788 (5.6)	702 (33.6)	
**Surgical Intervention (%)**			
**No Surgery**	6293 (8.8)	4441 (54.1)	<0.001
**Partial Nephrectomy**	22733 (31.8)	164 (2.0)	
**Radical Nephrectomy/Cytoreductive Nephrectomy**	42424 (59.4)	3598 (43.9)	
**Received Targeted Therapy (%)**	1365 (1.9)	3871 (47.0)	<0.001

For the 31,453 cM0 patients who underwent primary surgical treatment and had FG available, further stratification into high and low-risk cM0 RCC was completed, as described previously (Supplementary Table 1B). While both groups had similar rates of surgical intervention, patients with high-risk cM0 disease predominantly underwent RN, while those with low-risk disease were equally likely to receive RN or PNx. Median follow-up was similar between the groups.

### De novo metastatic (cM1) RCC and targeted therapy

**Figure 1:**
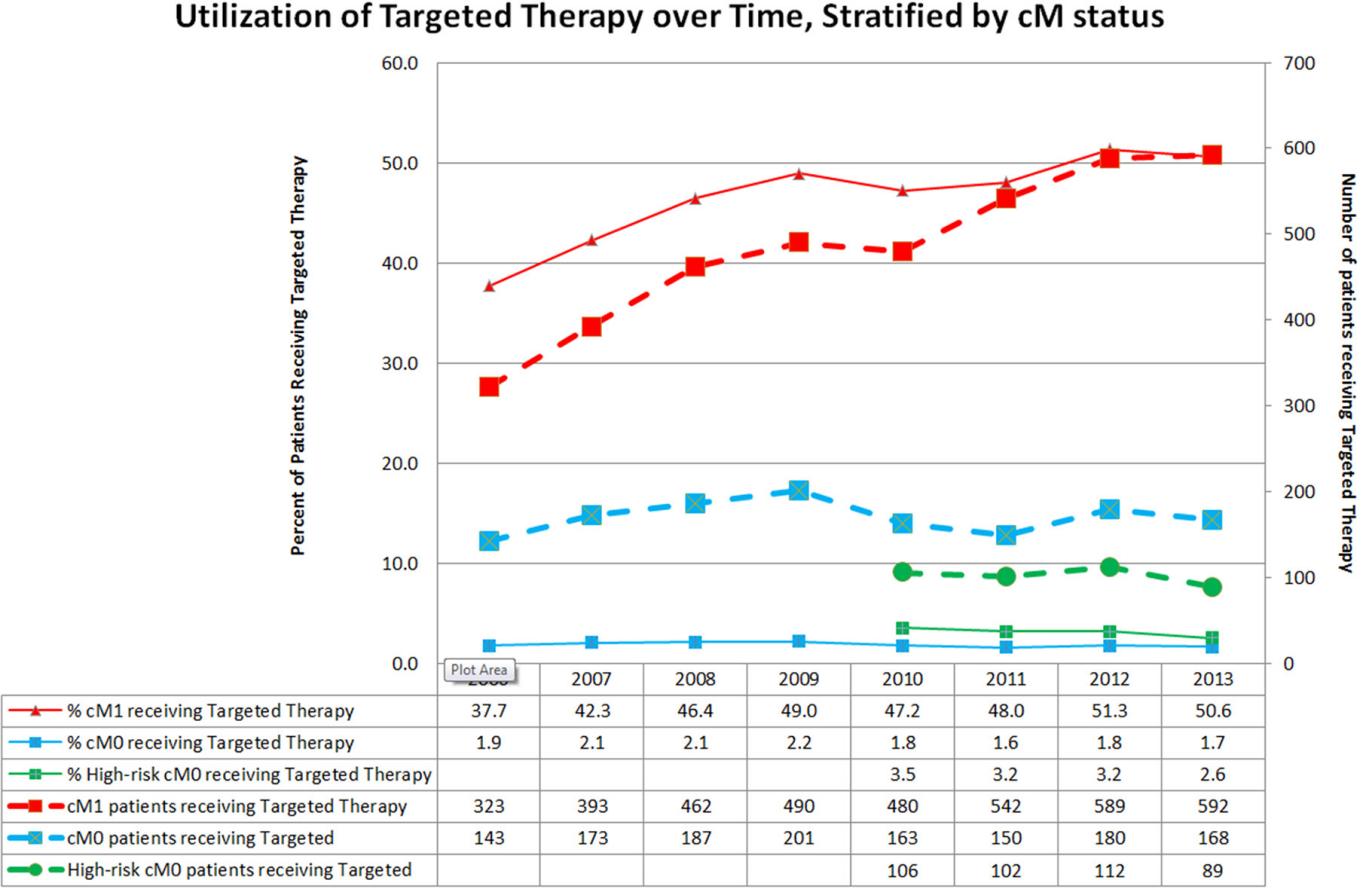
Utilization of targeted therapy over time, stratified by cM status

From 2006 to 2013, the proportion of eligible patients receiving targeted therapy steadily increased (Figure 1).

**Figure 2:**
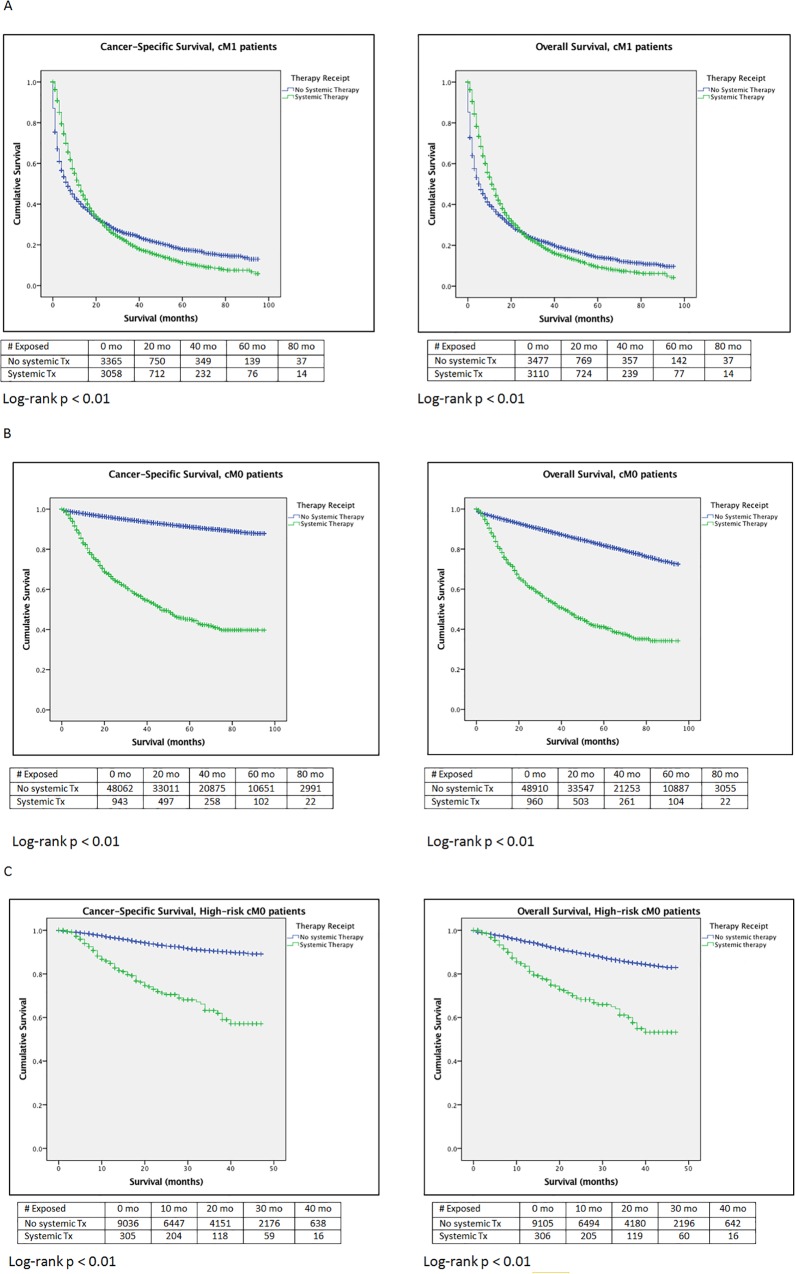
Kaplan–Meier survival analyses **(A)** Cancer-specific Survival and Overall Survival, cM1 patients (Entire cohort). **(B)** Cancer-specific Survival and Overall Survival, cM0 patients (Entire cohort). **(C)** Cancer-specific Survival and Overall Survival, High-risk cM0 patients.

Table 2A highlights the predictors of TT receipt in this population. Higher cT-stage and presence of nodal disease at diagnosis increased TT utilization, while surgical intervention (RN or PNx) decreased it. Older patients, patients in the lowest SES quartile, uninsured or Medicaid patients, and single or widowed patients were less likely to receive TT. Histology of the primary tumor did not impact TT receipt in this population. On competing risks analysis assessing predictors of CSM (Supplementary Table 2B), the use of TT was associated with reduced CSM. Higher cT-stage, cN+ disease, and papillary and sarcomatoid histology were the strongest predictors of increased CSM, but older age, being widowed, and treatment in the Midwestern US were also associated with increased CSM. Surgical intervention drastically reduced CSM. In a subset analysis of patients with only clear cell histology (Supplementary Table 2C), the use of TT and surgical intervention were still associated with reduced CSM. In a subset analysis of cM1 patients who received TT (not shown), surgical intervention with RN or PNx was associated with reduced CSM (HR 0.42 and 0.47, respectively; p<0.01). Figure 2A depicts the CSS and OS of patients with cM1 disease, with the majority of events occurring within the first 20 months regardless of treatment. In untreated patients, the 5-year CSS and OS was 18% and 14%, respectively; in treated patients, the 5-year CSS and OS were 13% and 11%, respectively.

**Table 2A:** Predictors of targeted therapy receipt in cM1 patients, multivariable logistic regression analysis

	*OR*	*95% CI for OR*		*p-Value*
***Lower***	***Higher***
***Age at Diagnosis***	0.97	0.96	0.97	<0.01
***Sex***				
***Male***	Reference			
***Female***	0.95	0.85	1.06	0.37
***Race***				
***Non-Hispanic White***	Reference			
***Hispanic***	1	0.86	1.17	0.99
***Native American***	2.18	1.31	3.62	<0.01
***Asian or Pacific Islander***	1.2	0.96	1.5	0.11
***Non-Hispanic Black***	0.81	0.68	0.97	0.02
***Socioeconomic Status***				
***First Quartile (highest)***	Reference			
***Second Quartile***	0.85	0.72	1	0.04
***Third Quartile***	0.84	0.72	0.98	0.03
***Fourth Quartile (lowest)***	0.78	0.66	0.91	<0.01
***Insurance***				
***Insured***	Reference			
***Medicaid***	0.79	0.68	0.92	<0.01
***Uninsured***	0.69	0.54	0.88	<0.01
***Region***				
***Northeast***	Reference			
***Southeast***	1.08	0.89	1.3	0.43
***Midwest***	1.18	0.98	1.42	0.07
***West***	0.98	0.83	1.16	0.84
***Marital Status***				
***Married***	Reference			
***Single***	0.63	0.55	0.73	<0.01
***Divorced/Separated***	0.97	0.83	1.13	0.66
***Widowed***	0.71	0.59	0.84	<0.01
***Laterality***				
***Bilateral primary***	Reference			
***Left-sided primary***	1.11	0.55	2.25	0.77
***Right-sided primary***	1.12	0.55	2.27	0.75
***cT stage***				
***cT1***	Reference			
***cT2***	1.28	1.1	1.49	<0.01
***cT3***	1.28	1.12	1.47	<0.01
***cT4***	1.4	1.18	1.66	<0.01
***cN stage***				
***cN0***	Reference			
***cN1***	1.46	1.31	1.62	<0.01
***Histology***				
***Clear cell RCC***	Reference			
***Papillary RCC***	0.88	0.69	1.12	0.31
***Chromophobe RCC***	0.76	0.45	1.3	0.32
***Sarcomatoid RCC***	0.93	0.75	1.15	0.48
***Surgical Intervention***				
***No Surgery***	Reference			
***Partial Nephrectomy***	0.4	0.28	0.59	<0.01
***Radical Nephrectomy***	0.73	0.65	0.83	<0.01

### Non-metastatic (cM0) RCC and targeted therapy

As TTs have not yet been approved in the adjuvant setting, TT utilization in patients with non-metastatic cM0 RCC are presumed to represent salvage therapy for recurrent disease. Between 2006 and 2013, 1.6-2.2% of all cM0 patients received targeted therapy annually, while in the high-risk cM0 subset, 2.5-3.5% of patients received targeted therapy (Figure 1).

Amongst all cM0 patients, higher cT-stage and presence of nodal disease were the strongest predictors of TT receipt (Table 3A). Surgically untreated patients were more likely to receive TT than patients who underwent RN or PNx. While patients with sarcomatoid histology were more likely to receive TT compared to patients with clear cell histology, patients with chromophobe RCC were less likely. Similar to the cM1 cohort, older patients, patients in the lowest SES quartile and single or divorced patients were less likely to receive TT. Hispanic patients were also less likely to receive TT than non-Hispanic whites. In the high-risk cM0 subset, older age and disease factors drove TT receipt (Supplementary Table 3B).

**Table 3A:** Predictors of targeted therapy receipt in cM0 patients, multivariable logistic regression analysis

	*OR*	*95% CI for OR*		*p Value*
***Lower***	***Higher***
***Age at Diagnosis***	0.97	0.97	0.98	<0.01
***Sex***				
***Male***	Reference			
***Female***	0.89	0.78	1.03	0.11
***Race***				
***Non-Hispanic White***	Reference			
***Hispanic***	0.81	0.65	0.99	0.04
***Native American***	0.58	0.26	1.31	0.19
***Asian or Pacific Islander***	1.2	0.92	1.57	0.18
***Non-Hispanic Black***	0.79	0.63	1	0.05
***Socioeconomic Status***				
***First Quartile (highest)***	Reference			
***Second Quartile***	0.85	0.7	1.04	0.11
***Third Quartile***	0.91	0.75	1.09	0.3
***Fourth Quartile (lowest)***	0.73	0.6	0.89	<0.01
***Insurance***				
***Insured***	Reference			
***Medicaid***	0.86	0.69	1.07	0.18
***Uninsured***	0.71	0.49	1.03	0.07
***Region***				
***Northeast***	Reference			
***Southeast***	1.04	0.83	1.31	0.71
***Midwest***	0.8	0.64	1.01	0.06
***West***	0.96	0.79	1.17	0.68
***Marital Status***				
***Married***	Reference			
***Single***	0.76	0.63	0.92	0.01
***Divorced/Separated***	0.87	0.7	1.07	0.19
***Widowed***	0.76	0.59	0.98	0.03
***Laterality***				
***Bilateral primary***	Reference			
***Left-sided primary***	1.01	0.25	4.08	0.99
***Right-sided primary***	0.9	0.22	3.63	0.88
***cT stage***				
***cT1***	Reference			
***cT2***	3.91	3.2	4.78	<0.01
***cT3***	9.16	7.75	10.84	<0.01
***cT4***	18.67	14	24.91	<0.01
***cN stage***				
***cN0***	Reference			
***cN1***	5.7	4.79	6.78	<0.01
***Histology***				
***Clear cell RCC***	Reference			
***Papillary RCC***	0.99	0.8	1.23	0.93
***Chromophobe RCC***	0.56	0.39	0.79	<0.01
***Sarcomatoid RCC***	2.7	2	3.64	<0.01
***Surgical Intervention***				
***No Surgery***	Reference			
***Partial Nephrectomy***	0.05	0.04	0.07	<0.01
***Radical Nephrectomy***	0.15	0.13	0.19	<0.01

Amongst the entire cM0 cohort, higher cT-stage, cN+ disease and sarcomatoid histology were most predictive of increased CSM (Table 4A). While surgical intervention was still associated with reduced CSM, TT receipt was associated with increased CSM. Older age, male sex, uninsured or Medicaid coverage, treatment in the Southeast, and marital status (divorced/separated or widowed) were also associated with increased CSM. On subset analysis of high-risk cM0 patients (Supplementary Table 4B), TT receipt was associated with increased CSM. Amongst the high-risk cM0 patients with only clear cell histology, TT receipt was still associated with increased CSM (Supplementary Table 4C).

**Table 4A:** Predictors of cancer-specific mortality in cM0 patients, fine and gray competing risk proportional hazards regressions analysis

	*HR*	*95% CI for HR*		*p Value*
		*Lower*	*Higher*	
***Age at Diagnosis***	1.03	1.03	1.04	<0.01
***Sex***				
***Female***	Reference			
***Male***	1.1	1.02	1.19	0.01
***Race***				
***Non-Hispanic White***	Reference			
***Hispanic***	1	0.9	1.1	0.92
***Native American***	1.01	0.71	1.43	0.97
***Asian or Pacific Islander***	1.07	0.92	1.25	0.36
***Non-Hispanic Black***	1.08	0.96	1.22	0.22
***Socioeconomic Status***				
***First Quartile (highest)***	Reference			
***Second Quartile***	0.96	0.86	1.07	0.47
***Third Quartile***	1.1	0.98	1.23	0.1
***Fourth Quartile (lowest)***	1.08	0.97	1.2	0.16
***Insurance***				
***Insurance***	Reference			
***Medicaid***	1.2	1.05	1.37	0.01
***Uninsured***	1.25	1.02	1.53	0.03
***Region***				
***Northeast***	Reference			
***Southeast***	1.18	1.04	1.33	0.01
***Midwest***	1.03	0.91	1.18	0.61
***West***	1.02	0.92	1.14	0.67
***Marital Status***				
***Married***	Reference			
***Single***	1.12	0.97	1.27	0.08
***Divorced/Separated***	1.19	1.07	1.32	<0.01
***Widowed***	1.22	1.1	1.37	<0.01
***Laterality***				
***Right-sided primary***	Reference			
***Left-sided primary***	1	0.93	1.07	0.99
***Bilateral primary***	1.88	0.9	3.9	0.09
***cT stage***				
***cT1***	Reference			
***cT2***	2.77	2.52	3.05	<0.01
***cT3***	4.51	4.15	4.89	<0.01
***cT4***	8.26	6.53	10.46	<0.01
***cN stage***				
***cN0***	Reference			
***cN1***	3.64	3.18	4.16	<0.01
***Histology***				
***Clear cell RCC***	Reference			
***Papillary RCC***	1	0.88	1.13	0.97
***Chromophobe RCC***	0.47	0.38	0.58	<0.01
***Sarcomatoid RCC***	4.13	3.4	5.03	<0.01
***Surgical Intervention***				
***No surgery***	Reference			
***Partial Nephrectomy***	0.13	0.11	0.15	<0.01
***Radical Nephrectomy***	0.33	0.29	0.37	<0.01
***Receipt of Targeted Therapy***				
***No/Unknown***	Reference			
***Yes***	1.84	1.58	2.14	<0.01

Figure 2B and 2C depict the CSS and OS of all patients with cM0 disease and the high-risk cM0 subset, respectively. Amongst the entire cM0 cohort, patients that never received targeted therapy had a 91% 5-year CSS and 82% 5-year OS. In treated patients in the entire cM0 cohort, 5-year CSS and OS were 45% and 41%, respectively. In the high-risk cM0 cohort over a 4-year period, untreated patients had 89% CSS and 82% OS, while treated patients had 55% CSS and 51% OS.

## DISCUSSION

The introduction of TTs has drastically changed the RCC treatment paradigm, specifically for metastatic disease. With improvements in progression-free survival in the range of 3-8 months, [6, 7, 16] they have become a cornerstone of therapy for metastatic RCC [1, 2, 17, 18]. However, with that success, there has been a significant effort to utilize these therapies in the adjuvant setting for high-risk localized or locally advanced RCC. Two randomized controlled trials provided conflicting evidence for its utility, [12, 15] and consensus is still pending regarding the appropriateness in this setting. While other groups have assessed survival outcomes in the cytokine era, [19–21] we utilized the SEER database to evaluate trends of TT utilization, particularly in the non-metastatic setting, thereby adding some context for the discussion regarding the benefit of adjuvant therapy.

For contrast, de novo metastatic RCC patients were evaluated first. The use of TTs for these patients has steadily increased since 2006, though they remain underutilized [17, 18, 21–24]. Their use provided cancer-specific survival benefit in the entire cM1 cohort and in the clear-cell subset (Supplementary Table 2B and 2C), and the primary benefit appears to be within the first 20 months (Figure 2A). While not unsurprising on its own, this data serves as an important comparison when assessing the use and efficacy of TTs in the non-metastatic setting.

Of note, while the benefit of cytoreductive nephrectomy was established in the cytokine era, [9] many retrospective studies have attempted to address the role of cytoreductive nephrectomy in the TT era [8, 11, 25]. In this study, in patients with cM1 disease who received TT, cytoreductive surgery significantly reduced CSM, suggesting continued benefit. However, without data regarding timing of TT in relation to surgery and patient-specific comorbidities, it is difficult to determine the specific benefit of cytoreductive surgery in the TT era.

In the cM0 patients, although the SEER database does not provide information regarding timing of TT in relation to surgery, due to lack of FDA approval, it is presumed that the primary utilization of TT was as salvage therapy for metastatic disease. Utilization trends are consistent with this, as utilization ranged between 1.6-2.2% during this 8-year time period. Even amongst high-risk cM0 patients, classified based on ASSURE and S-TRAC criteria, utilization was limited (2.6-3.5%). TT receipt was associated with an increased CSM in the entire cM0 cohort, high-risk cM0 cohort and the high-risk clear cell cM0 cohort (HR 1.65-1.84) (Table 4A and Supplementary Table 4C). While likely due to selection bias for patients with worse pathology and progression to metastatic disease, it highlights the fact that patients receiving targeted therapy have poor prognosis due to their disease. However, KM curves for these cohorts (Figure 2B and 2C) highlight a few significant trends. First, the large proportions of cM0 patients that never received or required TT were more likely to die of other causes than RCC. Competing risks for other-cause mortality can significantly limit the utility of TT in a large unselected population. Additionally, the small population of patients treated with TT in these cohorts fared much better than patients with cM1 disease at diagnosis.

This large dataset provides some key contributions to the discussion of adjuvant therapy for RCC. Five-year disease progression (recurrence, second malignancy, death) in the placebo arms of ASSURE and S-TRAC were 36.8% and 48.7%, respectively. However, the strict selection bias for clinical trials limits their generalizability [12, 15]. In our cohort, 2-3% of the cM0 population received TT. As such, providing adjuvant therapy for the entire population or even the entire high-risk cM0 population could result in overtreatment in 97-98% of patients. Even accounting for underutilization, recurrence rates for cM0 patients managed surgically have been reported to be 10-20% [26]. As these targeted therapies are known to have significant adverse events, [27] this would subject a large population to unnecessary significant toxicity.

From a survival benefit standpoint, the large proportion of patients cM0 patients who never progress to needing salvage therapy are likely to die of non-RCC related causes rather than RCC itself. The small proportion that do progress to metastatic disease and are initiated on targeted therapy still have better CSS and OS than patients diagnosed with de novo metastatic disease, suggesting that salvage therapy is still a viable option with survival benefit.

Our study has clear limitations, including the lack of granularity in the SEER database as it relates to the TT agents, therapy sequencing and timing, patient comorbidities, and timing of clinical progression. This precludes our ability to analyze the subset of cM0 patients who did progress to metastatic disease, which could enable better selection of patients that may benefit from adjuvant therapy. Additionally, we are unable to identify the specific TT utilized, and traditional chemotherapeutic agents may potentially be included in the TT analysis; however, as RCC has been known to be chemo-resistant, the incidence is likely to be low during the TT era. Prior studies have demonstrated very low rates of chemotherapy use prior to the introduction of targeted therapies [17, 18, 21]. Lastly, as it relates to the chemotherapy variable, prior comparison to SEER-Medicare datasets have demonstrated 65-80% sensitivity, but very high specificity [28]. As such, the SEER registries specifically list utilization as “Yes” or “No/Unknown,” which we adhere to in the included analyses. It is possible, therefore, that utilization rates in our study underestimate true utilization by 25-50%. However, with utilization rates as low as they are, this would increase utilization to approximately 5% even in the high-risk cohort.

Despite its limitations, this study is the largest population-based study to assess the use of TT in the management of RCC. By evaluating its utilization and survival impact in both the de novo metastatic and cM0 setting, we demonstrate the potential effect of using TTs as adjuvant therapy in a more generalizable setting. Specifically, unlike the prior RCTs, we highlight the potential over-treatment of cM0 patients who are unlikely to ever need TT and the survival benefit afforded by salvage therapy. In an unscreened and unselected population, competing risks for other-cause mortality may limit the utility of adjuvant therapy in cM0 RCC. Our results reinforce the findings of the ASSURE trial, [12, 14] and thereby provides population-based support for not using TT in an adjuvant setting.

## MATERIALS AND METHODS

### Study population

The Surveillance, Epidemiology, and End Results (SEER) database reports cancer-specific outcomes from specific geographic areas representing 28% of the US population. Using the SEER database, we identified patients with five RCC-specific histology codes and the kidney as primary tumor site. Patients receiving radiotherapy prior to therapy were excluded (N=335). Only patients diagnosed after 2006 were included to capture the targeted therapy era [3, 5].

### Description of covariates

Demographic variables of interest included age at diagnosis, gender, race, insurance status, marital status, and region based on SEER registry (West, Northeast, Southeast, Midwest). Based on prior literature, [29] a county-level socioeconomic (SES) measure was created, based on the percentage of individuals (i) with less than a high school education, (ii) below the poverty line, (iii) unemployed, (iv) foreign-born, and (v) median household income. Disease-specific covariates included clinical stage (T-stage [cT], N-stage [cN], and M-stage [cM]), primary histology type, histologic grade (2010-2013 only), surgical intervention (radical nephrectomy or partial nephrectomy), and receipt of targeted therapy.

While TTs, including sunitinib, sorafenib, temsirolimus, everolimus, pazopanib, axitinib, and cabozantinib, are captured by the SEER chemotherapy variable, immunotherapies (bevacizumab, IL-2, immune checkpoint inhibitors) are not.

### Statistical analysis

Patients were stratified by cM status (cM0 vs cM1), and cM0 patients who underwent primary surgery were subsequently risk stratified. As Furhman Grade (FG), an important component of the “high-risk” designation for localized RCC, is only available from 2010-2013, only these patients were included in this subset analysis. Based on a combination of the ASSURE and S-TRAC clinical trials, [12, 15] the following patients were considered high-risk cM0 RCC: cT1-2 & FG 3-4, cT3 & FG 2-4, any cT4 disease, and cN1 disease. All other cM0 patients who underwent primary surgery in the 2010-2013 cohort were classified as low-risk cM0.

Descriptive statistics for demographic and socioeconomic variable comparisons were performed by the Student t-test for continuous variables and the chi-square test for categorical variables. Multivariable logistic regression models were performed to generate odds ratios (ORs) for the identification of factors associated with receipt of targeted therapy. Fine and Gray competing risks proportional regression modeling, censoring for non-cancer mortality, was performed to generate hazards ratios (HR) to identify predictors of cancer-specific mortality (CSM). Given the sample size and appropriate number of events, our variable selection was exploratory in nature, including all variables in the regression models. A subset analysis of clear cell RCC histology patients and high-risk cM0 patients was also performed. Kaplan–Meier analysis (log-rank test) was used to evaluate overall survival (OS) and cancer-specific survival (CSS) for the same cohorts, stratified based on receipt of targeted therapy. All statistical tests were two-tailed and a p-value <0.05 was considered statistically significant. Statistical tests were performed using R statistical package - R Core Team (2012) and SAS 9.4 (SAS Institute, Cary, North Carolina).

### Key message

Population-level analyses highlight the low utilization of targeted therapy in cM0 patients and the potential overtreatment that could result from an adjuvant therapy paradigm in RCC. Competing risks for other-cause mortality make the real-world utility of adjuvant targeted therapy limited.

## SUPPLEMENTARY MATERIALS FIGURES AND TABLES




