# Supplementary material for: High competing risks minimize real-world utility of adjuvant targeted therapy in renal cell carcinoma: a population-based analysis
Source: Oncotarget. 2018 Mar 30;9(24):16731–43. doi: 10.18632/oncotarget.24675 (PMC5908282; doi:10.18632/oncotarget.24675)
Supplement: Supplementary file 1 [file oncotarget-09-16731-s001.pdf]

High competing risks minimize real-world utility of adjuvant targeted therapy in renal cell carcinoma: a population-based analysis

SUPPLEMENTARY MATERIALS

Supplementary Table 1B: Patient Demographic of cM0 patients by Risk Stratification

See Supplementary File 1

Supplementary Table 2B: Predictors of Cancer-Specific Mortality in cM1 patients, Fine and Gray competing risk proportional hazards regressions analysis

See Supplementary File 1

Supplementary Table 2C: Predictors of Cancer-Specific Mortality in cM1 patients with Clear Cell Histology, Fine and Gray competing risk proportional hazards regressions analysis

See Supplementary File 1

Supplementary Table 3B: Predictors of Targeted Therapy Receipt in the High-risk cM0 population, Multivariable Logistic Regression Analysis

See Supplementary File 1

Supplementary Table 4B: Predictors of Cancer-Specific Mortality in High-risk cM0 patients, Fine and Gray competing risk proportional hazards regressions analysis

See Supplementary File 1

Supplementary Table 4C: Predictors of Cancer-Specific Mortality in High-risk cM0 patients with Clear Cell RCC Histology, Fine and Gray competing risk proportional hazards regressions analysis

See Supplementary File 1
