# Supplementary material for: High competing risks minimize real-world utility of adjuvant targeted therapy in renal cell carcinoma: a population-based analysis
Source: Oncotarget. 2018 Mar 30;9(24):16731–43. doi: 10.18632/oncotarget.24675 (PMC5908282; doi:10.18632/oncotarget.24675)
Supplement: Supplementary file 2 [file oncotarget-09-16731-s002.pdf]

TABLE 1B [SUPPLEMENT]
Patient Demographic of cM0 patients by Risk Stratification

	Low-Risk cM0	High-Risk cM0	p-value
Total Number, N	18328	13125	
Age at Diagnosis, Mean (SD)	59.63 (12.49)	62.16 (12.15)	<0.001
Sex, Male (%)	10939 (59.7)	8965 (68.3)	<0.001
Region (%)			
Southeast	3396 (18.5)	2292 (17.5)	<0.001
Midwest	3011 (16.4)	2347 (17.9)	
West	9022 (49.2)	6663 (50.8)	
Northeast	2899 (15.8)	1823 (13.9)	
Insurance (%)			
Medicaid	1869 (10.4)	1284 (9.9)	0.123
Uninsured	534 (3.0)	426 (3.3)	
Insured	15601 (86.7)	11254 (86.8)	
Marital Status (%)			
Single	2843 (16.5)	1907 (15.4)	0.004
Divorced/Separated	1906 (11.1)	1314 (10.6)	
Widowed	1311 (7.6)	1037 (8.4)	
Married	11179 (64.8)	8159 (65.7)	
Race (%)			
Hispanic	2709 (14.8)	1764 (13.4)	0.019
American Indian/Alaskan	157 (0.9)	108 (0.8)	
Asian or Pacific Islander	930 (5.1)	690 (5.3)	
Black	2111 (11.5)	1494 (11.4)	
White	12314 (67.2)	9003 (68.6)	
Socioeconomic Status (%)			
1 = Highest quartile	3295 (18.0)	2538 (19.3)	<0.001
2	4100 (22.4)	2715 (20.7)	
3	4944 (27.0)	3544 (27.0)	
4 = Lowest quartile	5989 (32.7)	4328 (33.0)	
Laterality (%)			
Right-sided primary	9375 (51.2)	6570 (50.1)	0.142
Left-sided primary	8942 (48.8)	6543 (49.9)	
Bilateral	6 (0.0)	6 (0.0)	
Histology (%)			
Clear Cell RCC	12447 (67.9)	8616 (65.6)	<0.001
Papillary RCC	2607 (14.2)	1791 (13.6)	
Chromophobe RCC	859 (4.7)	734 (5.6)	
Sarcomatoid RCC	25 (0.1)	190 (1.4)	
RCC, Unspecified	2390 (13.0)	1794 (13.7)	
cT stage (%)			
cT1	16293 (88.9)	6084 (46.4)	<0.001
cT2	1836 (10.0)	1494 (11.4)	
cT3	199 (1.1)	5343 (40.7)	
cT4	0 (0.0)	204 (1.6)	
cN stage (%),			
CN1	0 (0.0)	507 (3.9)	<0.001
Fuhrman Grade (%)			
Grade 1	3413 (18.6)	5 (0.0)	<0.001
Grade 2	14915 (81.4)	2174 (16.7)	
Grade 3	0 (0.0)	9099 (69.7)	

Grade 4	0 (0.0)	1776 (13.6)	
Surgical Intervention (%)			
Partial Nephrectomy	9088 (49.6)	3702 (28.2)	<0.001
Radical Nephrectomy	9240 (50.4)	9423 (71.8)	
Received Targeted Therapy (%)	47 (0.3)	409 (3.1)	<0.001

TABLE 2B [SUPPLEMENT]

Predictors of Cancer-Specific Mortality in cM1 patients, Fine and Gray competing risk proportional hazards regressions analysis

	p Value	HR	95% CI for HR	
			Lower	Higher
Age at Diagnosis	<0.01	1.01	1.00	1.01
Sex				
Female	Reference			
Male	0.26	0.96	0.90	1.03
Race				
Non-Hispanic White	Reference			
Hispanic	0.12	0.93	0.85	1.02
Native American	0.03	0.76	0.59	0.97
Asian or Pacific Islander	0.04	0.86	0.75	0.99
Non-Hispanic Black	0.96	1.00	0.90	1.11
Socioeconomic Status				
First Quartile (highest)	Reference			
Second Quartile	0.05	1.11	1.00	1.22
Third Quartile	0.08	1.09	0.99	1.19
Fourth Quartile (lowest)	<0.01	1.19	1.08	1.30
Insurance				
Insurance	Reference			
Medicaid	0.71	1.02	0.93	1.12
Uninsured	0.36	0.93	0.79	1.09
Region				
Northeast	Reference			
Southeast	0.24	1.07	0.96	1.20
Midwest	<0.01	1.24	1.11	1.38
West	0.30	1.06	0.95	1.17
Marital Status				
Married	Reference			
Single	0.27	1.05	0.96	1.15
Divorced/Separated	0.86	0.99	0.90	1.09
Widowed	0.01	1.15	1.04	1.28
Laterality				
Right-sided primary	Reference			
Left-sided primary	0.68	1.01	0.96	1.07
Bilateral primary	0.26	0.81	0.55	1.18
cT stage				
cT1	Reference			
cT2	<0.01	1.21	1.10	1.33
cT3	<0.01	1.44	1.32	1.57
cT4	<0.01	1.61	1.45	1.79
cN stage				
cN0	Reference			
cN1	<0.01	1.50	1.41	1.60
Histology				
Clear cell RCC	Reference			
Papillary RCC	0.03	1.17	1.02	1.34
Chromophobe RCC	0.65	0.93	0.69	1.27
Sarcomatoid RCC	<0.01	2.24	2.01	2.50
Surgical Intervention				
No surgery	Reference			

Partial Nephrectomy	<0.01	0.34	0.26	0.43
Radical Nephrectomy	<0.01	0.38	0.36	0.41
Receipt of Targeted Therapy				
No/Unknown	Reference			
Yes	<0.01	0.73	0.69	0.77

TABLE 2C [SUPPLEMENT]

Predictors of Cancer-Specific Mortality in cM1 patients with Clear Cell Histology, Fine and Gray competing risk proportional hazards regressions analysis

	p Value	*HR*	*95% CI for HR*	
			Lower	*Higher*
Age at Diagnosis	<0.01	1.01	1.00	1.01
Sex				
Female		Reference		
Male	0.02	0.89	0.81	0.99
Race				
Non-Hispanic White		Reference		
Hispanic	0.97	1.00	0.87	1.16
Native American	0.09	0.62	0.35	1.07
Asian or Pacific Islander	0.90	1.01	0.84	1.22
Non-Hispanic Black	0.02	1.25	1.04	1.50
Socioeconomic Status				
First Quartile (highest)		Reference		
Second Quartile	0.64	1.04	0.90	1.20
Third Quartile	0.35	1.07	0.93	1.22
Fourth Quartile (lowest)	0.15	1.10	0.96	1.27
Insurance				
Insured		Reference		
Medicaid	0.63	0.96	0.82	1.13
Uninsured	0.35	0.89	0.69	1.14
Region				
Northeast		Reference		
Southeast	0.69	1.04	0.87	1.24
Midwest	0.15	1.13	0.96	1.35
West	0.60	0.96	0.82	1.13
Marital Status				
Married		Reference		
Single	<0.01	1.22	1.06	1.40
Divorced/Separated	0.60	1.04	0.90	1.20
Widowed	0.63	1.05	0.87	1.25
Laterality				
Right-sided primary		Reference		
Left-sided primary	0.73	1.02	0.93	1.11
Bilateral primary	0.31	0.57	0.19	1.67
cT stage				
cT1		Reference		
cT2	<0.01	1.30	1.12	1.52
cT3	<0.01	1.45	1.26	1.66
cT4	<0.01	1.76	1.48	2.10
cN stage				
cN0		Reference		
cN1	<0.01	1.73	1.56	1.92
Surgical Intervention				
No surgery		Reference		
Partial Nephrectomy	<0.01	0.32	0.23	0.45
Radical Nephrectomy	<0.01	0.37	0.33	0.41
Receipt of Targeted Therapy				
No/Unknown		Reference		
Yes	<0.01	0.87	0.79	0.95

TABLE 3B [SUPPLEMENT]

Predictors of Targeted Therapy Receipt in the High-risk cM0 population, Multivariable Logistic Regression Analysis

	p Value	*HR*	*95% CI for HR*	
			Lower	*Higher*
Age at Diagnosis	<0.01	0.98	0.97	0.99
Sex				
Female		Reference		
Male	0.17	1.20	0.92	1.55
Race				
Non-Hispanic White		Reference		
Hispanic	0.26	0.81	0.56	1.17
Native American	1.00	NA	NA	NA
Asian or Pacific Islander	0.50	1.17	0.74	1.86
Non-Hispanic Black	0.37	0.82	0.53	1.27
Socioeconomic Status				
First Quartile (highest)		Reference		
Second Quartile	0.46	0.88	0.63	1.23
Third Quartile	0.40	0.87	0.63	1.20
Fourth Quartile (lowest)	0.30	0.84	0.60	1.17
Insurance				
Insured		Reference		
Medicaid	0.09	0.70	0.46	1.06
Uninsured	0.08	0.51	0.25	1.07
Region				
Northeast		Reference		
Southeast	0.12	0.73	0.49	1.09
Midwest	0.05	0.67	0.45	1.00
West	0.39	0.87	0.62	1.20
Marital Status				
Married		Reference		
Single	0.19	0.80	0.57	1.12
Divorced/Separated	0.13	0.74	0.50	1.10
Widowed	0.03	0.55	0.31	0.95
Laterality				
Bilateral primary		Reference		
Left-sided primary	0.20	0.20	0.02	2.30
Right-sided primary	0.28	0.26	0.02	3.02
cT stage				
cT1		Reference		
cT2	<0.01	3.24	1.94	5.40
cT3	<0.01	7.99	5.30	12.04
cT4	<0.01	22.19	12.44	39.59
cN stage				
cN0		Reference		
cN1	<0.01	5.13	3.82	6.90
Histology				
Clear cell RCC		Reference		
Papillary RCC	0.93	1.02	0.69	1.50
Chromophobe RCC	0.01	0.40	0.19	0.82
Sarcomatoid RCC	0.04	1.76	1.03	3.01

Fuhrman Grade	0.12	NA	NA	NA
Surgical Intervention				
Radical Nephrectomy	Reference			
Partial Nephrectomy	<0.01	0.47	0.31	0.71

TABLE 4B [SUPPLEMENT]

Predictors of Cancer-Specific Mortality in High-risk cM0 patients, Fine and Gray competing risk proportional hazards regressions analysis

	p Value	HR	95% CI for HR	
			Lower	Higher
Age at Diagnosis	<0.01	1.02	1.02	1.03
Sex				
Female	Reference			
Male	0.71	0.97	0.81	1.15
Race				
Non-Hispanic White	Reference			
Hispanic	0.48	0.91	0.70	1.18
Native American	0.69	0.80	0.28	2.27
Asian or Pacific Islander	0.18	1.25	0.90	1.74
Non-Hispanic Black	0.01	1.39	1.08	1.79
Socioeconomic Status				
First Quartile (highest)	Reference			
Second Quartile	0.38	0.89	0.69	1.16
Third Quartile	0.45	1.09	0.87	1.38
Fourth Quartile (lowest)	0.71	0.96	0.75	1.21
Insurance				
Insured	Reference			
Medicaid	0.50	1.10	0.84	1.43
Uninsured	0.74	1.07	0.70	1.64
Region				
Northeast	Reference			
Southeast	0.74	0.95	0.70	1.29
Midwest	0.67	1.07	0.79	1.43
West	0.91	1.02	0.78	1.33
Marital Status				
Married	Reference			
Single	0.87	0.98	0.76	1.26
Divorced/Separated	0.02	1.35	1.06	1.71
Widowed	0.16	1.22	0.92	1.62
Laterality				
Right-sided primary	Reference			
Left-sided primary	0.69	1.03	0.89	1.20
Bilateral primary	NA	NA	NA	NA
cT stage				
cT1	Reference			
cT2	<0.01	2.00	1.47	2.71
cT3	<0.01	3.46	2.71	4.42
cT4	<0.01	11.05	7.73	15.79
cN stage				
cN0	Reference			
cN1	<0.01	3.37	2.69	4.22
Histology				
Clear cell RCC	Reference			
Papillary RCC	0.70	1.06	0.80	1.393
Chromophobe RCC	<0.01	0.42	0.26	0.671
Sarcomatoid RCC	<0.01	2.91	2.13	3.971

Fuhrman Grade				
Grade 2	Reference			
Grade 3	<0.01	2.33	1.78	3.052
Grade 4	<0.01	5.45	4.13	7.198
Surgical Intervention				
Radical Nephrectomy	Reference			
Partial Nephrectomy	<0.01	0.45	0.32	0.63
Receipt of Targeted Therapy				
No/Unknown	Reference			
Yes	<0.01	1.65	1.28	2.13

TABLE 4C [SUPPLEMENT]

Predictors of Cancer-Specific Mortality in High-risk cM0 patients with Clear Cell RCC Histology, Fine and Gray competing risk proportional hazards regressions analysis

	p Value	HR	95% CI for HR	
			Lower	Higher
Age at Diagnosis	<0.01	1.03	1.02	1.04
Sex				
Female		Reference		
Male	0.27	0.88	0.70	1.10
Race				
Non-Hispanic White		Reference		
Hispanic	0.50	0.90	0.65	1.23
Native American	0.53	1.43	0.47	4.37
Asian or Pacific Islander	0.08	1.42	0.96	2.10
Non-Hispanic Black	0.09	1.40	0.95	2.06
Socioeconomic Status				
First Quartile (highest)		Reference		
Second Quartile	0.80	0.96	0.69	1.33
Third Quartile	0.28	1.17	0.88	1.57
Fourth Quartile (lowest)	0.52	1.10	0.82	1.48
Insurance				
Insured		Reference		
Medicaid	0.59	0.91	0.65	1.28
Uninsured	0.94	0.98	0.56	1.72
Region				
Northeast		Reference		
Southeast	0.75	0.93	0.62	1.41
Midwest	0.99	1.00	0.68	1.47
West	0.63	0.92	0.64	1.31
Marital Status				
Married		Reference		
Single	0.45	1.13	0.83	1.53
Divorced/Separated	0.00	1.55	1.15	2.08
Widowed	0.42	1.17	0.81	1.70
Laterality				
Right-sided primary		Reference		
Left-sided primary	0.48	1.07	0.88	1.31
Bilateral primary	NA	NA	NA	NA
cT stage				
cT1		Reference		
cT2	<0.01	2.08	1.41	3.07
cT3	<0.01	3.26	2.39	4.44
cT4	<0.01	12.82	8.02	20.48
cN stage				
cN0		Reference		
cN1	<0.01	2.95	2.10	4.15
Fuhrman Grade				
Grade 2		Reference		
Grade 3	<0.01	2.03	1.49	2.77
Grade 4	<0.01	5.12	3.73	7.04
Surgical Intervention				

Radical Nephrectomy	Reference			
Partial Nephrectomy	<0.01	0.45	0.29	0.69
Receipt of Targeted Therapy				
No/Unknown	Reference			
Yes	<0.01	1.74	1.24	2.43
